# Complete genome sequence of *Thermotoga* sp. strain RQ7

**DOI:** 10.1186/s40793-017-0271-1

**Published:** 2017-10-11

**Authors:** Zhaohui Xu, Rutika Puranik, Junxi Hu, Hui Xu, Dongmei Han

**Affiliations:** 1 Department of Biological Sciences, Bowling Green State University, Bowling Green, OH 43403 USA; 2 School of Life Sciences, Minnan Normal University, 36 Xianqianzhi Street, Zhangzhou, Fujian 363000 China

**Keywords:** *Thermotoga*, *T.* sp. strain RQ7, Natural competence, CRISPR, Restriction-modification system, TneDI, CP007633

## Abstract

*Thermotoga* sp. strain RQ7 is a member of the family *Thermotogaceae* in the order *Thermotogales*. It is a Gram negative, hyperthermophilic, and strictly anaerobic bacterium. It grows on diverse simple and complex carbohydrates and can use protons as the final electron acceptor. Its complete genome is composed of a chromosome of 1,851,618 bp and a plasmid of 846 bp. The chromosome contains 1906 putative genes, including 1853 protein coding genes and 53 RNA genes. The genetic features pertaining to various lateral gene transfer mechanisms are analyzed. The genome carries a complete set of putative competence genes, 8 loci of CRISPRs, and a deletion of a well-conserved Type II R-M system.

## Background

10.1601/nm.459 species are a group of thermophilic or hyperthermophilic bacteria that can ferment a wide range of carbohydrates and produce hydrogen gas as one of the major final products [1, 2]. Their hydrogen yield from glucose can reach the theoretical maximum: 4 mol of H_2_ from each mole of glucose [2, 3], which makes them ideal candidates for biofuel production. Meanwhile, because their enzymes are thermostable by nature, they also hold great prospect in the biocatalyst sector. 16S rRNA gene sequence analyses place 10.1601/nm.459 at a deep branch in the tree of life, and genomic studies also reveal extensive horizontal gene transfer events between 10.1601/nm.457 and other groups, particularly *Archaea* and 10.1601/nm.3874 [4]. Controversy over the phylogenetic significance of 10.1601/nm.459 has triggered a prolonged debate on the concepts of species and biogeography, etc. [5].

We have been interested in the genetics of 10.1601/nm.459 over the years and have developed the earliest set of tools to genetically modify these bacteria [6–8]. Strain RQ7 plays an essential role in these studies. This strain possesses the smallest known plasmid, pRQ7 (846 bp) [9], that is absent from most 10.1601/nm.459 strains and serves as the base vector for all *Thermotoga-E. coli* shuttle vectors developed so far. *T*. sp. strain RQ7 is also the first 10.1601/nm.459 strain in which natural competence was discovered [7]. To gain insights into the genetic and genomic features of the strain and to facilitate the continuing effort on developing genetic tools for 10.1601/nm.459, we set out to sequence the whole genome of *T.* sp. strain RQ7.

## Organism information

### Classification and features

**Fig. 1:**
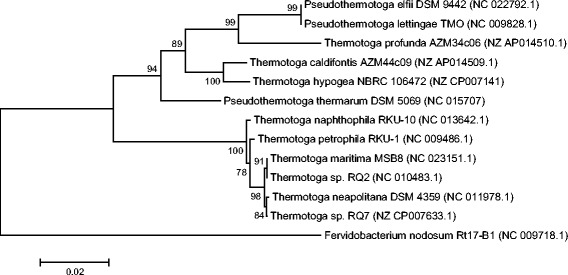
Phylogenetic tree showing the position of *T.* sp. strain RQ7 relative to other species within the order *Thermotogales*. Only species with complete genome sequences are included. The tree was built with 16S rRNA gene sequences, using the Neighbor-Joining method with MEGA7 [50]. *Fervidobacterium nodosum* serves as the outgroup

*T.* sp. strain RQ7 was isolated from marine sediments of Ribeira Quente, Azores [1]. The strain is a member of the genus 10.1601/nm.459 *,* the family 10.1601/nm.458, and the order 10.1601/nm.457 (Table 1). Based on 16S rRNA gene sequences, the closest relative of *T.* sp. strain RQ7 is 10.1601/nm.465 10.1601/strainfinder?urlappend=%3Fid%3DDSM+4359 *,* and these two strains cluster with 10.1601/nm.460 MSB8 and *T*. sp. strain RQ2 (Fig. 1). The results are in agreement with previous reports [10].

**Table 1:** Classification and general features of *Thermotoga sp.* strain RQ7 according to the MIGS recommendations [36]

MIGS ID	Property	Term	Evidence code^a^
	Classification	Domain *Bacteria*	TAS [37]
		Phylum *Thermotogae*	TAS [38, 39]
		Class *Thermotogae*	TAS [39, 40]
		Order *Thermotogales*	TAS [39, 41]
		Family *Thermotogaceae*	TAS [39, 42]
		Genus *Thermotoga*	TAS [1, 43, 44]
		Species *T. neapolitana*	IGC, TSA [45, 46]
		strain: RQ7	TAS [1]
	Gram stain	Negative	TAS [1]
	Cell shape	Rod	IDA, TAS [1]
	Motility	Motile	IDA, TAS [1]
	Sporulation	Not reported	
	Temperature range	55–90 °C	TAS [1]
	Optimum temperature	Around 80 °C	TAS [1]
	pH range; Optimum	5.5–9; 6.5	IDA, TAS [1]
	Carbon source	Mono- and polysaccharides	IDA, TAS [1, 47, 48]
MIGS-6	Habitat	Geothermally heated sediments	TAS [1]
MIGS-6.3	Salinity	0.25–3.75% NaCl (*w*/*v*)	IDA, TAS [1]
MIGS-22	Oxygen requirement	Anaerobic	IDA, TAS [1]
MIGS-15	Biotic relationship	Free-living	IDA, TAS [1]
MIGS-14	Pathogenicity	Non-pathogen	IDA, TAS [1]
MIGS-4	Geographic location	Azores, Sao Miguel, Ribeira Quente	TAS [1]
MIGS-5	Sample collection	1985	NAS
MIGS-4.1	Latitude	Not reported	
MIGS-4.2	Longitude	Not reported	
MIGS-4.4	Altitude	About sea level	NAS

^a^Evidence codes - *IDA* Inferred from Direct Assay, *TAS* Traceable Author Statement (i.e., a direct report exists in the literature), *NAS* Non-traceable Author Statement (i.e., not directly observed for the living, isolated sample, but based on a generally accepted property for the species, or anecdotal evidence), *IGC* Inferred from Genomic Content (i.e., average nucleotide identity, syntenic regions). These evidence codes are from the Gene Ontology project [49]

**Fig. 2:**
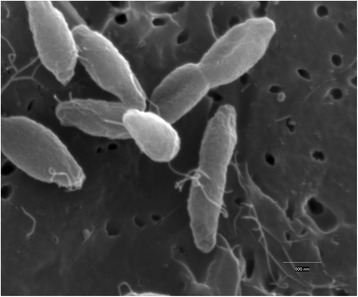
Scanning electron micrograph of *T.* sp. strain RQ7 cells after 12 h of growth. Bar, 0.5 μm

Like its close relatives 10.1601/nm.465 10.1601/strainfinder?urlappend=%3Fid%3DDSM+4359 and 10.1601/nm.460 MSB8, *T.* sp. strain RQ7 is a strict anaerobe, growing best around 80 °C, utilizing both simple and complex sugars, and producing hydrogen gas. These bacteria grow in both rich and defined media, are free living and non-pathogenic to humans, animals, or plants. Cells are rod-shaped, about 0.5 to 2 μm in length and 0.4 to 0.5 μm in diameter (Fig. 2). The most distinctive feature of 10.1601/nm.459 cells is the “toga” structure that balloons out from both ends of the rod [1, 11], an extension of their outer membrane [12].

## Genome sequencing information

### Genome project history

The project started in June 2011, and the genome was sequenced by BGI Americas (Cambridge, MA) using the Illumina technology. A total of 400 Mb of clean data were generated, which covered the genome more than 200 fold. The assembled scaffold covers 97.7% of the chromosome. PCR and Sanger sequencing were later used for gap filling. The assembly was finalized in February 2014, and the complete sequence was submitted to the GenBank in April 2014. The sequence was annotated with the NCBI Prokaryotic Genome Annotation Pipeline [13] and the DOE-JGI Microbial Genome Annotation Pipeline (MGAP v.4) [14]. The project information is summarized in Table 2.

**Table 2:** Project information

MIGS ID	Property	Term
MIGS 31	Finishing quality	Complete
MIGS-28	Libraries used	Three Illumina paired-end libraries in sizes of 500, 2000, and 5000 bp
MIGS 29	Sequencing platforms	Illumina and Sanger
MIGS 31.2	Fold coverage	> 200×
MIGS 30	Assemblers	SOAPdenovo [17], SOAPaligner [18], CLC Workbench 5.1 [19], and GapFish [20]
MIGS 32	Gene calling method	GeneMarkS+ [51], Prodigal [52]
	Locus Tag	TRQ7 in GenBank; Ga0077854 in JGI-IMG
	GenBank ID	CP007633, KF798180
	GenBank Date of Release	February 4, 2015
	GOLD ID	Gp0117593
	BIOPROJECT	PRJNA246218
MIGS 13	Source Material Identifier	Personal culture collection (Dr. Harald Huber)
	Project relevance	Bioenergy, biotechnology, evolution

### Growth conditions and genomic DNA preparation

*T.* sp. strain RQ7 was kindly provided by Drs. Harald Huber and Robert Huber at the University of Regensburg, Germany. It was cultivated in SVO medium [15] at 77 °C, and its genomic DNA was extracted with standard phenol extraction method [16]. Briefly, cells from 250 ml of overnight culture were collected by centrifugation and resuspended in 10 ml of STE solution (10 mM Tris-HCl, 1 mM EDTA, 100 mM NaCl, pH 8.0). SDS and proteinase K were added to a final concentration of 1% (*w*/*v*) and 20 μg/ml. The mixture was incubated at 50 °C for 6 h followed by the addition of an equal volume of phenol/chloroform/isoamyl alcohol (25:24:1, *v*/v/v). After gentle mixing, the mixture was centrifuged at 12,000 g at 4 °C for 15 min. The upper aqueous layer was transferred to a clean tube and mixed with 1/10 volume of 3 M sodium acetate (pH 5.5) and 2 volumes of ice cold 95% (v/v) ethanol. The DNA was spooled out by a glass rod, washed with 70% (v/v) ethanol, air dried, dissolved in 2 ml of TE buffer (10 mM Tris-HCl, 1 mM EDTA, pH 8.0) containing 20 μg/ml RNase A, and stored at −20 °C.

### Genome sequencing and assembly

The genome of *T.* sp. strain RQ7 was mainly sequenced by BGI Americas using Illumina HiSeq 2000 sequencing platform. Three paired-end libraries, in size of 500, 2000, and 5000 kb, were constructed. The raw data were filtered by a quality control step and generated 400 Mb of clean data, which indicated a coverage of more than 200-fold. The reads were assembled by SOAPdenovo [17] and polished by SOAPaligner [18]. This resulted in a single scaffold of 1,822,593 bp that covered 97.7% of the genome and contained 28 gaps. The gap filling efforts included the integration of the current scaffold with contigs generated by the CLC Genomics Workbench [19] and a small amount of public sequences in GenBank. GapFish [20] was then used to solve a dozen ambiguous regions. Finally, PCR and primer walking were performed to close the remaining gaps, resulting a final assembly of 1,851,618 bp. The entire assembling process integrated wet lab methods with in silico approaches, and the programs used included public software (SOAPdenovo and SOAPaligner [17, 18]), a commercial product (CLC Genomics Workbench [19]), and an in-house program GapFish [20]. Details of the assembling process are described in our previous report [20].

### Genome annotation

The genome was independently annotated by two pipelines, the NCBI Prokaryotic Genome Annotation Pipeline [13] and the DOE-JGI Microbial Genome Annotation Pipeline (MGAP v.4) [14]. Both pipelines combine a gene-calling algorithm with a similarity-based gene detection approach, even though the algorithms and databases they use are different. For example, PGAAP uses GeneMarkS+ for de novo gene prediction, while MGAP uses Prodigal. Consequently, the two pipelines produced slightly different annotation results. The analyses in this report took into consideration of the results from both pipelines and are assisted with manual curation.

## Genome properties

**Fig. 3:**
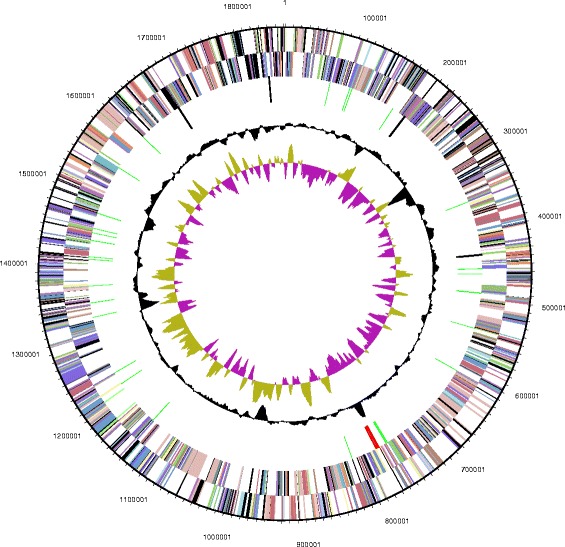
Chromosomal map of *T.* sp. strain RQ7. From outside to the center: genes on forward strand (color by COG categories), genes on reverse strand (color by COG categories), RNA genes (tRNAs: green, rRNAs: red, other RNAs: black), GC content (black), GC skew (olive/purple)

The genome of *T*. sp. strain RQ7 is composed of a circular chromosome of 1,851,618 bp with a GC content of 47.05% and a single mini-plasmid of 846 bp with a GC percentage of 39.95 (Fig. 3; Table 3). The plasmid pRQ7 has been characterized [9] and sequenced [6, 21] before. According to the annotation of MGAP, the chromosome carries 1906 putative genes, of which, 1853 are protein coding genes and 53 are RNA genes (Table 4). Among all the genes that are assigned to a COG category (Table 5), a significant portion (~12%, 191 genes) are devoted to carbohydrate utilization, which is typical to 10.1601/nm.459 strains and accords with their versatile use of carbon and energy sources.

**Table 3:** Summary of genome: one chromosome and one plasmid

Label	Size (bp)	Topology	INSDC identifier	RefSeq ID
Chromosome	1,851,618	Circular	CP007633	NZ_CP007633
pRQ7	846	Circular	KF798180	NC_023152

**Table 4:** Genome statistics according to the MGAP pipeline annotation (chromosome only)

Attribute	Value	% of total
Genome size (bp)	1,851,618	100.00
DNA coding (bp)	1,768,561	95.51
DNA G + C (bp)	871,250	47.05
DNA scaffolds	1	
Total genes	1906	100.00
Protein coding genes	1853	97.22
RNA genes	53	2.78
Pseudo genes	–	–
Genes in internal clusters	110	5.77
Genes with function prediction	1522	79.85
Genes assigned to COGs	1453	76.23
Genes with Pfam domains	1629	85.47
Genes with signal peptides	35	1.84
Genes with transmembrane helices	462	24.24
CRISPR repeats	8

**Table 5:** Number of genes associated with general COG functional categories

Code	Value	%age	Description
J	165	10.17	Translation, ribosomal structure and biogenesis
A	–	–	RNA processing and modification
K	75	4.62	Transcription
L	53	3.27	Replication, recombination and repair
B	1	0.06	Chromatin structure and dynamics
D	19	1.17	Cell cycle control, Cell division, chromosome partitioning
V	34	2.09	Defense mechanisms
T	57	3.51	Signal transduction mechanisms
M	74	4.56	Cell wall/membrane biogenesis
N	55	3.39	Cell motility
U	21	1.29	Intracellular trafficking and secretion
O	66	4.07	Posttranslational modification, protein turnover, chaperones
C	104	6.41	Energy production and conversion
G	191	11.77	Carbohydrate transport and metabolism
E	169	10.41	Amino acid transport and metabolism
F	65	4	Nucleotide transport and metabolism
H	73	4.5	Coenzyme transport and metabolism
I	42	2.59	Lipid transport and metabolism
P	103	6.35	Inorganic ion transport and metabolism
Q	18	1.11	Secondary metabolites biosynthesis, transport and catabolism
R	156	9.61	General function prediction only
S	75	4.62	Function unknown
–	453	23.77	Not in COGs

The total is based on the total number of protein coding genes in the genome as annotated by MGAP v.4 [14]

## Insights from the genome sequence

The chromosomal sequence of *T.* sp. strain RQ7 was compared to those of 10.1601/nm.460 MSB8, 10.1601/nm.465 10.1601/strainfinder?urlappend=%3Fid%3DDSM+4359 *,* and *T*. sp. strain RQ2, with emphases on the genetic elements that have the highest impacts on genetic engineering attempts, such as natural competence genes, CRISPRs, and R-M systems.

### Full genome comparison

**Fig. 4:**
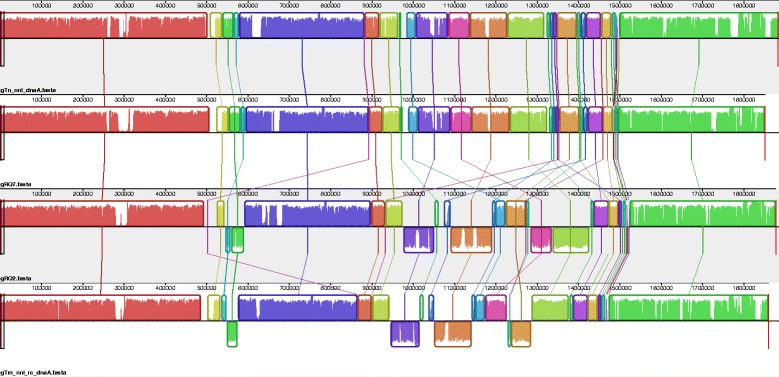
Full genome alignment of the four *Thermotoga* strains using Mauve [53]. Each horizontal panel represents one genome sequence, from top to bottom: *T. neapolitana* DSM 4359, *T.* sp. strain RQ7, *T.* sp. strain RQ2, and *T. maritima* MSB8. The sequences were downloaded from GenBank, and genomes of *T. neapolitana* DSM 4359 and *T. maritima* MSB8 were re-linearized at the *dnaA* gene. Blocks with the same color represent homologous regions. Blocks below the center lines are inversed regions. Inside of each block, the height of the similarity profile corresponds to the average level of conservation of the local area

The alignment of the complete genomic sequence of the four 10.1601/nm.459 strains (Fig. 4) revealed high levels of synteny among their genomes, particularly within the pairs of *T.* sp. strain RQ7−*T. neapolitana* 10.1601/strainfinder?urlappend=%3Fid%3DDSM+4359 and *T*. sp. strain RQ2−*T. maritima* MSB8*.* This is in agreement with their placements in the phylogenetic tree (Fig. 1). The average nucleotide identity between *T.* sp. strain RQ7 and the type strain 10.1601/nm.465 10.1601/strainfinder?urlappend=%3Fid%3DDSM+4359 is 98.49%, which is higher than the conventional cutoff of 95% for species delineation [22]. Therefore, *T.* sp. strain RQ7 should be considered as a strain of 10.1601/nm.465, same as *T*. sp. strain RQ2 to 10.1601/nm.460 [23].

A detailed comparison of *T.* sp. strain RQ7 and 10.1601/nm.465 10.1601/strainfinder?urlappend=%3Fid%3DDSM+4359 found 100 genes belonging only to the former and 120 genes only to the latter. Some of these genes became unique because their counterparts in the other genome have mutated to a pseudogene. However, many of the unique genes seem to have been acquired via recent lateral gene transfer events. The putative functions of these genes are mainly associated to transportation and utilization of carbohydrates and nucleotides. The most notable gene clusters include TRQ7_01555-01655 (nucleotide metabolism), TRQ7_02675-02725 (carbohydrate metabolism), TRQ7_03440-03490 (arabinose metabolism), CTN_0026-0038 (synthesis of antibiotics), CTN_0236-0245 (carbohydrate metabolism), CTN_0355-0373 (ribose metabolism), CTN_1540-1554 (carbohydrate metabolism), and CTN_1602-1627 (ribose metabolism). Follow-up functional genomics studies are needed to validate the predictions on these gene functions and metabolic pathways.

### Natural competence

10.1601/nm.459 species are known to undergo lateral gene transfer events. One of the ways this could happen is via natural transformation. Natural competence has been established in *T.* sp. strain RQ7 [7] and *T.* sp. strain RQ2 [8]. Using experimentally characterized competence genes as references, we are able to identify the genes that might play a role in natural competence in 10.1601/nm.459 (Table 6). These genes are widely spread among bacterial genomes, and none of them are clustered into operons. This might imply a primitive form of natural competence that is shared by most, if not all, bacteria. Perhaps, most free-living bacteria are more or less naturally competent during some points of their life. The trick is to identify the right conditions under which the natural competence will be allowed to develop.

**Table 6:** Manually curated competence genes

RQ7	Gene name^a^	Putative function	Tn	Tm	RQ2
DNA uptake and translocation					
TRQ7_00110	*pilZ* (Pa, Vc)	Type IV pilus biogenesis and twitching motility [54–56]	CTN_1670	TM0905	TRQ2_0022
TRQ7_00455	*pilB* (Pa, Vc)	Type II secretion system (T2SS), Type IV fimbrial assembly NTPase [57–59]	CTN_1739	TM0837	TRQ2_0090
TRQ7_01410 TRQ7_04530 TRQ7_08710	*pilQ* (Nm, Tt)	Secretin, forms gated channel for extrusion of assembled pilin [60–62]	CTN_1450 CTN_1933 CTN_0604	TM1117 TM0088	TRQ2_1699 TRQ2_0859
TRQ7_04500	*pilC* (Ps, Ng)	Type II secretory pathway, component PulF / Type IV fimbrial assembly protein [63, 64]	CTN_0598	TM_0094	TRQ2_0853
TRQ7_05855	*pilD* (Vv,Ng)	Type IV prepilin peptidase, processes N-terminal leader peptides for prepilins [65–67]	CTN_0883	TM1696	TRQ2_1138
TRQ7_06260	*comEC* (Bs)	Putative channel protein, Transports DNA across the cell membrane [68, 69]	CTN_0965	TM1775	TRQ2_1049
TRQ7_07315	*comF* (Hi)	Phosphoribosyltransferase [70, 71]	CTN_1168	TM1584	TRQ2_1247
TRQ7_07650	*pilT* (Ng)	Motility protein [72]	CTN_1229	TM1362	TRQ2_1467
TRQ7_07980	*pilE* (Ng, Pa)	Type IV pilin; major structural component of Type IV pilus [73, 74]	CTN_1301	TM1271	TRQ2_1548
TRQ7_09065	*comEA* (Bs)	High affinity DNA-binding periplasmic protein [75–78]	CTN_1515	TM1052	TRQ2_1756
Post-translocation					
TRQ7_02260	*comM* (Hi)	Promotes the recombination of the donor DNA into the chromosome [79]	CTN_0158	TM0513	TRQ2_0424
TRQ7_03645	*dprA* (Hi)	DNA protecting protein [80, 81]	CTN_0436	TM0250	TRQ2_0698

^a^Gene names are given after the experimentally characterized genes of the species in parentheses. *Pa Pseudomonas aeruginosa, Vc Vibrio cholerae*, *Nm Neisseria meningitidis*, *Tt Thermus thermophilus*, *Ps Pseudomonas stutzeri*, *Ng Neisseria gonorrhoeae*, *Vv Vibrio vulnificus*, *Bs Bacillus subtilis*, *Hi influenza, RQ7 T.* sp. strain RQ7, *Tn T. neapolitana* DSM 4359*, Tm T. maritima* MSB8*, RQ2 T.* sp. strain RQ2

### CRISPRs

**Fig. 5:**
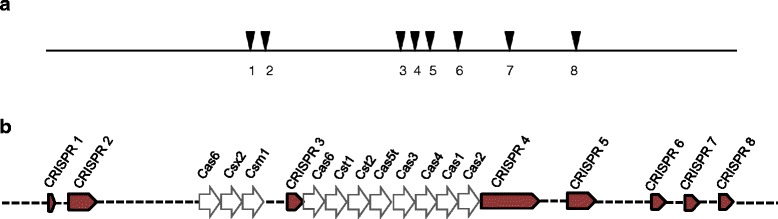
Diagrammatic representation of CRISPR/Cas systems in *T.* sp. strain RQ7. **a** Positions of the 8 regions of CRISPR arrays; drawn in scale using Clone Manager Professional Suite v.8 [82]. **b** Positions of the *cas* genes (open boxed) relative to the CRISPR arrays (filled boxes); not in scale

CRISPRs provide prokaryotes a form of adaptive immunity against invading phages and plasmids in a sequence specific manner [24, 25]. The system utilizes non-coding CRISPR RNA and a set of CRISPR-associated proteins to target invading nucleic acid, including both DNA and RNA. CRISPRs have been reported to prevent natural transformation [26, 27]. They have been noticed before in 10.1601/nm.459 and are credited for large scale chromosomal recombination events in these species [28, 29]. NCBI’s PGAAP pipeline identified 6 loci of CRISPR arrays in *T.* sp. strain RQ7, whereas JGI-IMG’s MGAP pipeline and a manual analysis using CRISPRFinder [30] recognized a total of 8 loci (Table 7). Among these eight CRISPR loci, #1 and #3 are the ones not considered by PGAAP. Two clusters of *cas* genes are also found. The *cas6-cas2* cassette is sandwiched between loci #3 and #4, and the *cas6-csm1* cassette is located 2285 bp upstream of locus #3 (Fig. 5, Table 7).

**Table 7:** Summary of CRISPR loci in *T.* sp. strain RQ7

Locus	Repeats	Coordinates^a^	No. of spacers	Cas genes
1	GTTTCAATCCTTCCTTAGAGGTATGGAAACA GTTTCAATACTTCCTTAGAGGTATGGAAACA GTTTCAATACTTCCTTTGAGGTATGAAAACA	553,849-554,014	2	No
2	TTTCCTATACCTCTAAGAAAGGATTGAAAC GTTTCCATACCTCTAAGGAAGTATTGAAAC	594,500-594,927	6	No
3	GTTTCAATACTTCCTTTGAGGTATGGAAA GTTTCAATACTTCCTTAGAGGTATGGAAA GTTTCAATACATCCTCAGAGGTATGATTT	975,191-975,420	3	Yes
4	GTTTTTATCTTCCTAAGAGGAATATGAAC GTTTTTATCTTCCTAAGAGGAATATAGTA	983,596-986,955	51	Yes
5	GTTTCAATACTTCCTTTGAGGTATGGAAAC GTTTCAATATTTCCTTATAGGTACAAACCC	1,011,410-1,012,101	10	No
6	GTTTCAATACTTCCTTAGAGGTATGGAAAC	1,090,312-1,090,681	5	No
7	GTTTCCATACCTCTAAGGAAGTATTGAAAC	1,233,649-1,233,878	3	No
8	GTTTCAATACTTCCTTTGAGGTATGGAAAC	1,422,811-1,423,509	10	No

^a^Coordinates as documented in JGI-IMG. The start coordinates in GenBank are 20 bp smaller because the chromosome is linearized at a site 20 bp downstream of what JGI-IMG uses

Although analysis with CRISPRFinder revealed the same number of CRISPR loci in the four close relatives, i.e. *T.* sp. strain RQ7, 10.1601/nm.465 10.1601/strainfinder?urlappend=%3Fid%3DDSM+4359 *,* 10.1601/nm.460 MSB8*,* and *T.* sp. strain RQ2, the total number of spacers they carry vary dramatically, as 95, 60, 106, and 129 spacers are found respectively. 10.1601/nm.460 MSB8 and *T.* sp. strain RQ2 also harbor RNA-targetting *cmr* genes in addition to DNA-targetting *cas* genes [31]. These differences may affect the efficiency of lateral gene transfer events among the strains.

### Type II R-M system TneDI

**Fig. 6:**
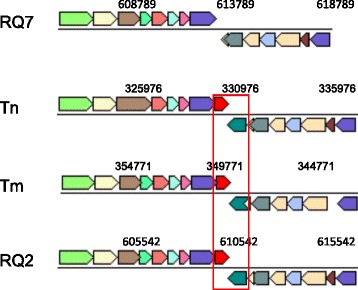
Deletion of the TneDI system in *T.* sp. RQ7. The neighborhoods of the deletion site were compared (color by COG categories). The big rectangle box highlights the R-M system that is absent in *T.* sp. strain RQ7 (show as RQ7 in the diagram). The numerical values are genome coordinates as documented in JGI-IMG. RQ2, *T.* sp. strain RQ2; Tm, *T. maritima* MSB8*;* Tn, *T. neapolitana* DSM 4359

**Fig. 7:**
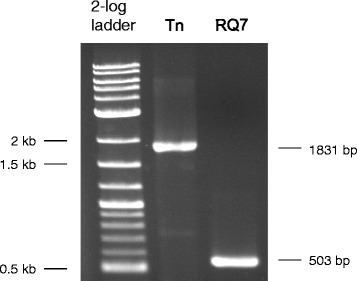
Experimental confirmation of the deletion of the TneDI system in *T.* sp. strain RQ7. *T. neapolitana* DSM 4359 (Tn) was used as the positive control. The expected sizes are 1831 bp in *T. neapolitana* DSM 4359 and 503 bp in *T.* sp. strain RQ7

**Fig. 8:**
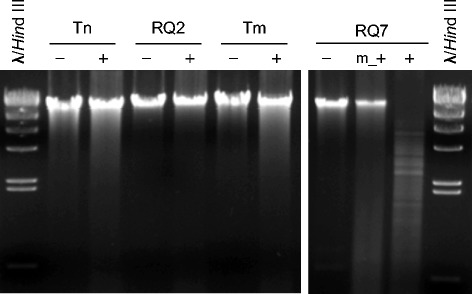
Digestion of the genomic DNA of *T. neapolitana* DSM 4359 (Tn), *T.* sp. strain RQ2 (RQ2), *T. maritima* MSB8 (Tm), and *T.* sp. strain RQ7 (RQ7) with R.TneDI. -, negative control, no R.TneDI; +, digestion with R.TneDI; m_+, DNA was treated with M.TneDI prior to being digested by R.TneDI

R-M systems are other defense mechanisms that prokaryotes have developed to protect the integrity of their genetic materials. The Type II R-M system TneDI has been characterized in 10.1601/nm.465 10.1601/strainfinder?urlappend=%3Fid%3DDSM+4359 and overexpressed in 10.1601/nm.3093 [32, 33]. The nuclease R.TneDI cleaves at the center of the recognition site (CG↓CG), and the methylase M.TneDI modifies one of the cytosines. The TneDI system has been found in many members of the 10.1601/nm.458 family, including 10.1601/nm.460 MSB8 and *T*. sp. strain RQ2 [32]. However, it is absent from *T.* sp. strain RQ7, although the neighborhood is still highly conserved (Fig. 6). To exclude the possibility of an assembling error, primers spanning the region in question were designed, and the PCR results confirmed the deletion (Fig. 7). The absence of the TneDI system makes the DNA of *T*. sp. strain RQ7 susceptible to R.TneDI, and in vitro treatment with M.TneDI provides complete protection to its genomic DNA (Fig. 8).

**Fig. 9:**
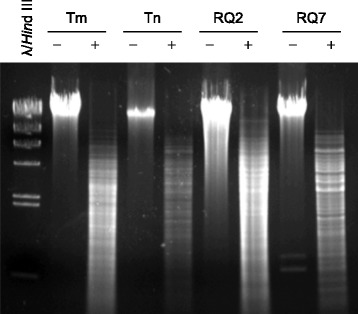
Digestion of genomic DNA of *T. maritima* MSB8 (Tm), *T. neapolitana* DSM 4359 (Tn), *T.* sp. strain RQ2 (RQ2), and *T.* sp. strain RQ7 by BstUI. -, negative control, no BstUI; +, treated with BstUI

M.TneDI has been predicted to be a m^4^C methylase based on sequence analysis [32]. It has also been noticed that m^4^C methylation is more common than m^5^C in thermophiles, probably due to a reduced risk of deamination [34]. The speculation of M.TneDI being a m^4^C methylase is further supported by the observation that the genomic DNA of TneDI-bearing species is still suspetible to BstUI (Fig. 9), which is an isoschizomer of R.TneDI and known to be blocked by m^5^C methylation [35].

## Conclusions

The genome of *T*. sp. strain RQ7 shares large regions of synteny with those of its close relatives, namely, 10.1601/nm.465 10.1601/strainfinder?urlappend=%3Fid%3DDSM+4359 *,* 10.1601/nm.460 MSB8, and *T*. sp. strain RQ2. They all have a complete set of putative competence genes, although natural transformation has yet to be established in 10.1601/nm.465 10.1601/strainfinder?urlappend=%3Fid%3DDSM+4359 and 10.1601/nm.460 MSB8. The same number of CRISPR loci are found in all four genomes, even though the number of spacers vary. The most noticeable difference among the strains is the absence of the TneDI R-M system in *T*. sp. strain RQ7, which partially explains why this strain is more amenable to genetic modifications than others. In general, this work sheds light on the genetic features of *T*. sp. strain RQ7, promoting genetic and genomic studies of 10.1601/nm.459 spp.

## Abbreviations

Cas: CRISPR associated
CRISPR: Clustered regularly interspaced short palindromic repeats
R-M: Restriction-modification
